# Nutritional Status and Tuberculosis Risk in Adult and Pediatric Household Contacts

**DOI:** 10.1371/journal.pone.0166333

**Published:** 2016-11-11

**Authors:** Omowunmi Aibana, Xeno Acharya, Chuan-Chin Huang, Mercedes C. Becerra, Jerome T. Galea, Silvia S. Chiang, Carmen Contreras, Roger Calderon, Rosa Yataco, Gustavo E. Velásquez, Karen Tintaya, Judith Jimenez, Leonid Lecca, Megan B. Murray

**Affiliations:** 1 Department of Internal Medicine, Division of General Internal Medicine, The University of Texas Health Science Center at Houston, McGovern Medical School, Houston, Texas, United States of America; 2 Department of Social and Environmental Health Research, London School of Hygiene & Tropical Medicine, London, United Kingdom; 3 Department of Anesthesiology, Perioperative and Pain Medicine, Brigham and Women’s Hospital, Boston, Massachusetts, United States of America; 4 Department of Global Health and Social Medicine, Harvard Medical School, Boston, Massachusetts, United States of America; 5 Partners In Health / Socios En Salud Sucursal Peru, Lima, Peru; 6 Department of Pediatrics, Alpert School of Medicine of Brown University, Providence, Rhode Island, United States of America; 7 Center for International Health Research, Rhode Island Hospital, Providence, Rhode Island, United States of America; 8 Department of Internal Medicine, Division of Infectious Diseases, Brigham and Women’s Hospital, Boston, Massachusetts, United States of America; McGill University, CANADA

## Abstract

**Background:**

Studies show obesity decreases risk of tuberculosis (TB) disease. There is limited evidence on whether high body mass index also protects against TB infection; how very high body mass indices influence TB risk; or whether nutritional status predicts this risk in children. We assessed the impact of body mass index on incident TB infection and disease among adults and children.

**Methods and Findings:**

We conducted a prospective cohort study among household contacts of pulmonary TB cases in Lima, Peru. We determined body mass index at baseline and followed participants for one year for TB infection and disease. We used Cox proportional regression analyses to estimate hazard ratios for incident TB infection and disease. We enrolled 14,044 household contacts, and among 6853 negative for TB infection and disease at baseline, 1787 (26.1%) became infected. A total of 406 contacts developed secondary TB disease during follow-up. Body mass index did not predict risk of TB infection but overweight household contacts had significantly decreased risk of TB disease (HR 0.48; 95% CI 0.37–0.64; p <0.001) compared to those with normal weight. Among adults, body mass index ≥ 35 kg/m^2^ continued to predict a lower risk of TB disease (HR 0.30; 95% CI 0.12–0.74; p 0.009). We found no association between high body mass index and TB infection or disease among children under 12 years of age.

**Conclusions:**

High body mass index protects adults against TB disease even at levels ≥ 35 kg/m^2^. This protective effect does not extend to TB infection and is not seen in children.

## Introduction

Obesity is now recognized as a chronic inflammatory state instigated and maintained by the production of bio-active adipocytokines, which co-modulate host metabolism and immune function [1]. Although the course of events that leads to this state is not completely elucidated, recent evidence implicates both innate and adaptive immune responses in the pathogenesis of “metaflammation,” defined as the low-grade, persistent inflammation induced by metabolic cells in response to excess caloric intake [2–4]. The impact of these alterations in immune responses on host susceptibility to infectious diseases appears to vary by specific pathogen and clinical syndrome; while obesity increases susceptibility to influenza, periodontitis and skin infections, there is conflicting data regarding obesity and increased risk of community acquired pneumonia or bacteremia [5]. Some evidence also suggests higher body mass index (BMI) may improve immune responses to certain infections; one study showed that immune reconstitution after initiation of antiretroviral therapy in HIV peaks at an optimal BMI between 25 and 30 kg/m^2^, with worse responses at higher and lower BMIs [6].

The host response to tuberculosis is unique among infectious diseases in that within the spectrum of overweight and obesity studied to date, there is an inverse linear relationship between BMI and the risk of TB disease [7]. Although epidemiologic studies attribute this effect to a reduction in disease progression [8–13], few have evaluated the association between BMI and TB *infection*, and it is thus unclear at which point in the infection/disease process protection occurs. If obesity leads to an exaggerated innate response to a mycobacterial challenge, it may result in early clearance of *Mycobacterium tuberculosis* (Mtb) as manifested by the absence of tuberculin reactivity. Conversely, a pro-inflammatory T-cell response may result in containment of an initial infection and abrogate disease progression.

The relationship between obesity and immune activation in TB is particularly interesting because of the current debate about the possible detrimental effect of exaggerated immune responses on TB susceptibility and phenotype. Both animal and human studies suggest that susceptibility to mycobacteria can result from either inadequate or excessive inflammation and that these extremes of host responses may be associated with distinct clinical presentations [14, 15]. According to this paradigm, classic adult pulmonary tuberculosis, with its characteristic cavitary lesions, represents an over-vigorous T-cell mediated response that leads to the development of necrotizing granulomas [16]. In contrast, AIDS patients and children under the age of two are more likely to present with disseminated and extra-pulmonary TB (EPTB) [17, 18]. Indeed, EPTB is often considered the hallmark of TB in the immunosuppressed [19].

The observation that obesity is an inflammatory state partly explains the consistent finding that higher BMI is associated with protection against TB but raises a number of questions unanswered to date. Given the expected impact of obesity on both innate and acquired immunity, is protection mediated by a reduction in infection after exposure, in progression after infection, or both? Given that excessive inflammation may exacerbate the development of classic cavitary TB, is there an optimal BMI above which protection is diminished? And finally, does obesity also reduce the risk of the TB disease phenotype typically associated with immunosuppression or are the mechanisms of disease progression in vulnerable groups like young children sufficiently different from that in adults that the protective effect of increased BMI no longer pertains? Here, we examine the impact of BMI on TB infection and disease progression among a cohort of child and adult contacts of TB patients.

## Materials and Methods

### Ethics Statement

The study was approved by the Institutional Review Board of Harvard School of Public Health and the Research Ethics Committee of the National Institute of Health of Peru. All study participants or guardians provided written informed consent.

### Setting and Study Design

We conducted this study in regions of Lima, Peru, where the TB incidence in 2013 was 165–192 cases per 100,000 persons. The study area includes 20 districts of metropolitan Lima comprising a population of approximately 3.3 million residents living in urban areas and peri-urban, informal shantytown settlements. Epidemiologic surveys of urban and shantytown populations in Lima confirm a high prevalence of chronic diseases and associated risk factors including overweight and obesity, DM, hypertension, binge drinking and smoking [20–22].

Between September 2009 and August 2012, we identified patients older than 15 years of age diagnosed with pulmonary TB (PTB) by the National TB Program (NTP) in 106 participating health centers. We ascertained whether these “index” TB patients had microbiologic confirmation of PTB disease with sputum smear and mycobacterial culture. Within two weeks of enrolling an index TB case, we visited the patient’s household to enroll all household contacts and screen for TB infection and disease. We assessed baseline TB infection status with the tuberculin skin test (TST) in those contacts who had no history of a positive TST or active TB disease. If contacts had TB symptoms or signs, we obtained sputum samples for smear and culture and referred them to a health center for evaluation and treatment according to Peru’s NTP guidelines [23]. Household contacts referred for evaluation were assessed by clinicians for pulmonary and extra-pulmonary TB disease as indicated. We offered HIV testing to all index cases and household contacts. At enrollment, we collected the following information: age, gender, height, weight, alcohol consumption, tobacco use, HIV status, self-reported diabetes mellitus (DM), comorbid disease (heart disease, high blood pressure, asthma, kidney disease, use of steroids or chemotherapy or immunosuppressant, any other self-reported chronic illness), number of Bacillus Calmette–Guérin (BCG) vaccination scars, initiation of isoniazid preventive therapy (IPT), history of TB disease, and housing asset (housing type, number of rooms, water supply, sanitation facilities, lighting, composition of exterior walls and floor and roof materials). We also documented index TB patients’ baseline smear status, the presence or absence of cavitary disease and tobacco and alcohol use. We re-evaluated contacts for TB disease at 2, 6 and 12 months after enrollment and performed repeat TST at 6 and 12 months for those who tested negative at baseline.

### Exposure and Outcome Definitions

We calculated BMI as weight in kilograms divided by the square of height in meters. For adult contacts ≥ 20 years, we defined baseline nutritional status as follows: underweight (BMI < 18.5 kg/m^2^), normal (BMI 18.5 –<25 kg/m^2^) and overweight (BMI ≥ 25 kg/m^2^). For children and adolescents < 20 years, we used WHO age and gender-specific BMI z-score tables to classify those with BMI z-score < –2 as underweight and those with z-score >2 as overweight [24]. Based on previously reported age-specific risks of TB disease after exposure [18, 25], we categorized contacts into the following age categories: <5, 5–11, 12–19, 20–49 and ≥ 50 years. We considered contacts to be heavy drinkers if they reported consuming ≥40 g or ≥3 alcoholic drinks daily. For each household, we derived a socioeconomic status (SES) score using principal components analysis of housing asset weighted by household size.

We classified contacts as having TB infection at baseline if they reported a history of TB disease or a positive TST, if their TST was ≥ 10 mm at enrollment, or if they were diagnosed with TB disease less than 15 days after enrollment of the index case. We considered contacts to have acquired TB infection during the study follow-up period if they were TB uninfected at baseline and later converted their TST or developed secondary TB disease. We considered contacts to have developed incident secondary TB disease if they had microbiologic confirmation by sputum smear or culture, if they had extra-pulmonary TB, or if they were clinically diagnosed by a physician and initiated on TB treatment at least 15 days after index case enrollment; we considered contacts to have co-prevalent TB disease if they were diagnosed less than 15 days after index case enrollment. We defined secondary TB disease among contacts younger than 18 years of age according to the consensus guidelines for classifying TB disease in children [26].

### Statistical Analysis

We excluded contacts of index cases who did not have microbiological confirmation of PTB and contacts with missing data on their height or weight. We evaluated the effect of nutritional status on the prevalence ratio of TB infection at baseline using a modified Poisson generalized estimating equation to account for correlation within households. We specified an exchangeable working correlation structure for observations within the same household and obtained empirical standard error estimates for inference. Follow-up time began for all participants from the date of study enrollment. For the outcome of TB infection, we considered only contacts who were uninfected at baseline. We defined the date of infection as the midpoint between date of enrollment and the date of positive TST result or diagnosis with secondary TB disease, and we censored contacts who remained TST negative at the date of last TST result. For incident TB disease, we excluded co-prevalent TB cases, and we censored contacts who were not diagnosed with TB disease at the date of death, loss to follow-up, or the end of study. We used Kaplan-Meier curves to evaluate event-free survival time and Cox frailty proportional hazards models to evaluate risk factors for incident TB infection and disease, accounting for clustering within households. We constructed univariate and multivariate models to separately evaluate the effect of baseline BMI on the risk of TB infection and disease during study follow-up. In the multivariate model, we included variables identified a priori as potential confounders (age, gender, heavy alcohol consumption, tobacco use, SES, HIV, DM and comorbid disease) and any others associated with the outcome with a p value < 0.2 in the univariate analysis. We verified the proportional hazards assumptions for each covariate by introducing an interaction term between the covariate and time; we stratified by variables for which the proportional hazards assumption did not hold.

We evaluated the interaction between age and nutritional status for both outcomes of TB infection and secondary TB disease, using the likelihood ratio test. To explore the effect of very high BMI on the risks of TB infection and disease, we conducted sensitivity analyses among adults aged 20 years and older further categorizing BMI as 30 –<35 kg/m^2^ and ≥ 35 kg/m^2^. We also conducted sensitivity analyses separately evaluating only those household contacts diagnosed with TB disease at least 90 days after index case enrollment, contacts who developed microbiologically confirmed PTB and those with EPTB.

## Results

**Fig 1:**
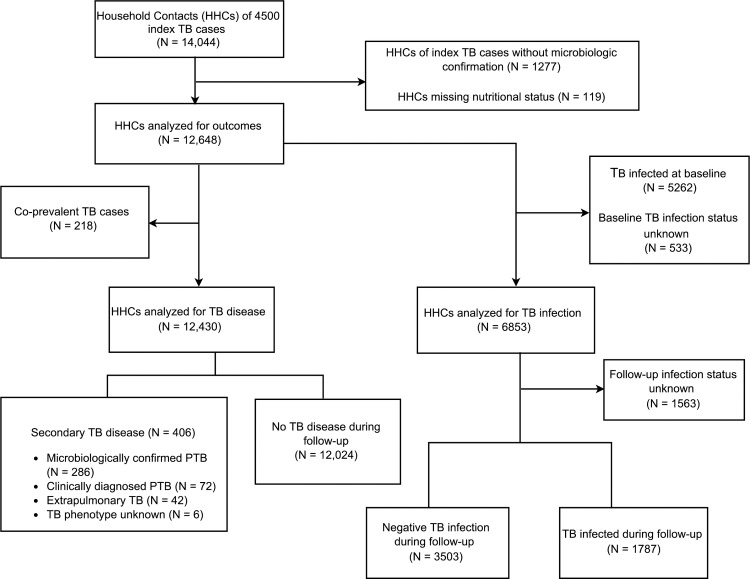
Flow diagram of household contacts of index tuberculosis (TB) cases.

We enrolled 14,044 household contacts of 4500 index TB cases. We excluded 1,277 (9.1%) contacts without a known microbiologically confirmed index case and 119 (0.8%) contacts whose baseline height or weight was missing (Fig 1). Of the remaining 12,648 contacts analyzed, 7310 (57.8%) were of normal weight, 5112 (40.4%) were overweight and 226 (1.8%) were underweight. Table 1 lists baseline characteristics of contacts according to nutritional status.

**Table 1:** Baseline characteristics of household contacts by nutritional status.

	Underweight (N = 226)	Normal weight (N = 7310)	Overweight (N = 5112)	Total (N = 12,648)	N^a^
	n (%)	n (%)	n (%)	n (%)	
**Household Contacts Characteristics**					
Age Categories					
<5 years	74 (32.7)	1280 (17.5)	271 (5.3)	1625 (12.9)	
5 to 11 years	39 (17.3)	1474 (20.2)	333 (6.5)	1846 (14.6)	
12 to 19 years	21 (9.3)	1757 (24.0)	192 (3.8)	1970 (15.6)	
20 to 49 years	70 (31.0)	2229 (30.5)	2918 (57.1)	5217 (41.3)	
≥50 years	22 (9.7)	570 (7.8)	1398 (27.4)	1990 (15.7)	
Male	110 (48.7)	3452 (47.2)	2101 (41.1)	5663 (44.8)	
Comorbid disease^b^	31 (13.7)	1148 (15.7)	1369 (26.8)	2548 (20.2)	12,645
Number of BCG Scars					12,645
0	43 (19.0)	1215 (16.6)	496 (9.7)	1754 (13.9)	
1	156 (69.0)	5021 (68.7)	2923 (57.2)	8100 (64.1)	
2	21 (9.3)	900 (12.3)	1318 (25.8)	2239 (17.7)	
> 2	6 (2.7)	172 (2.4)	374 (7.3)	552 (4.4)	
Isoniazid Preventive Therapy	77 (34.1)	2230 (30.5)	575 (11.3)	2882 (22.8)	12,639
Heavy Alcohol Use	7 (3.1)	300 (4.2)	357 (7.2)	664 (5.4)	12,393
Smokes	6 (2.7)	334 (4.6)	420 (8.3)	760 (6.1)	12,516
HIV Positive	5 (2.3)	27 (0.4)	19 (0.4)	51 (0.4)	12,499
Socioeconomic Status					12,316
Low	87 (39.2)	2523 (35.5)	1632 (32.7)	4242 (34.4)	
Middle	86 (38.7)	3240 (45.6)	2190 (43.9)	5516 (44.8)	
High	49 (22.1)	1342 (18.9)	1167 (23.4)	2558 (20.8)	
Self-Reported Diabetes Mellitus	0 (0.0)	53 (0.7)	168 (3.3)	221 (1.8)	12,555
History of TB	27 (12.0)	486 (6.7)	474 (9.3)	987 (7.8)	12,628
TB Infection at baseline	84 (39.6)	2645 (37.8)	2533 (51.73)	5262 (43.4)	12,115
**Index Case Characteristics**					
HIV Positive	11 (4.9)	235 (3.3)	178 (3.5)	424 (3.4)	12,518
Smear Positive	169 (74.8)	5584 (76.4)	3935 (77.0)	9688 (76.6)	12,641
Smear Grade among smear positive					9688
+	82 (48.5)	2594 (46.5)	1881 (47.8)	4557 (47.0)	
++	28 (16.6)	1116 (20.0)	776 (19.7)	1920 (19.8)	
+++	59 (34.9)	1874 (33.6)	1278 (32.5)	3211 (33.1)	
Cavitary Disease	55 (25.0)	1894 (26.4)	1381 (27.3)	3330 (26.7)	12,463
Tobacco use	7 (3.2)	196 (2.7)	140 (2.8)	343 (2.8)	12,404
Heavy Alcohol Use	27 (12.9)	727 (10.4)	471 (9.6)	1225 (10.1)	12,133

BCG, Bacillus Calmette–Guérin.

^a^ Total number of subjects with data for corresponding variable.

^b^ Comorbid disease includes any of the following: heart disease, high blood pressure, asthma, kidney disease, use of steroids or chemotherapy or immunosuppressant, any other self-reported chronic illness.

### Risk of TB infection

After we excluded 533 contacts whose baseline TB infection status was missing, 5262 of 12,115 contacts (43.4%) were found to be infected at baseline. In the univariate and adjusted analyses, overweight contacts were slightly more likely to have TB infection at baseline with RR 1.39 (95% CI 1.34–1.45; p < 0.0001) and adjusted RR 1.03 (95% CI 0.99–1.08; p 0.13) respectively (Table 2). Among the 6853 contacts who were negative for TB infection and disease at baseline (Fig 1), 1787 (26.1%) became infected during the 12-month follow up period. Table 3 shows that in the univariate analysis, overweight participants were 1.7 times as likely to become TB infected as those of normal weight (HR 1.71; 95% CI 1.54–1.90; p <0.001) but that when we adjusted for potential confounders, the difference did not reach statistical significance (HR 1.09; 95% CI 0.95–1.24). In a sensitivity analysis, we noted a trend toward increased risk of TB infection among adults aged 20 and older with BMI 30 –<35 kg/m^2^ and BMI ≥ 35 kg/m^2^ compared to those with normal weight (Table 4) but this was not statistically significant. We found no evidence of effect modification by age on the association between BMI and risk of TB infection (*p*-value for interaction 0.69).

**Table 2:** Univariate and multivariate adjusted risk of baseline tuberculosis (TB) infection.

	Univariate RR (95% CI)	p value	Multivariate RR (95% CI)	p value
**Household Contacts Characteristics**				
Age Categories				
< 5 years	0.31 (0.28–0.35)	<0.0001	0.32 (0.28–0.36)	<0.0001
5 to 11 years	0.62 (0.58–0.66)	<0.0001	0.65 (0.59–0.71)	<0.0001
12 to 19 years	0.91 (0.87–0.95)	<0.0001	0.90 (0.86–0.95)	<0.0001
20 to 49 years	0.48 (0.44–0.52)	<0.0001	0.50 (0.45–0.55)	<0.0001
≥50 years	Reference		Reference	
Male	0.92 (0.88–0.96)	<0.0001	0.98 (0.94–1.02)	0.32
Nutritional Status				
Underweight	1.06 (0.91–1.24)	0.44	1.15 (0.99–1.33)	0.07
Overweight	1.39 (1.34–1.45)	<0.0001	1.03 (0.99–1.08)	0.13
Normal	Reference		Reference	
Comorbid Disease^a^	1.17 (1.12–1.22)	<0.0001	1.01 (0.96–1.06)	0.74
Number of BCG Scars				
0	Reference		Reference	
1	1.06 (0.99–1.13)	0.07	1.01 (0.95–1.08)	0.71
2	1.48 (1.38–1.58)	<0.0001	1.10 (1.02–1.19)	0.01
> 2	1.67 (1.53–1.83)	<0.0001	1.22 (1.11–1.34)	<0.001
Isoniazid Preventive Therapy	0.60 (0.56–0.64)	<0.0001	1.00 (0.92–1.08)	0.94
Heavy Alcohol Use	1.35 (1.26–1.45)	<0.0001	1.10 (1.03–1.18)	0.01
Smokes	1.30 (1.21–1.39)	<0.0001	1.01 (0.95–1.09)	0.71
HIV Positive	1.25 (0.96–1.63)	0.10	0.99 (0.72–1.38)	0.97
Socioeconomic Status				
Middle	Reference		Reference	
Low	1.06 (1.00–1.13)	0.05	1.06 (1.00–1.13)	0.05
High	1.02 (0.95–1.09)	0.65	0.93 (0.86–0.99)	0.03
Self-Reported Diabetes Mellitus	1.38 (1.24–1.54)	<0.0001	1.11 (0.99–1.24)	0.07
**Index Cases Characteristics**				
HIV Positive	1.08 (0.93–1.25)	0.33	NA	
Smear Positive	1.14 (1.07–1.22)	<0.0001	1.11 (1.04–1.18)	0.002
Cavitary Disease	1.12 (1.05–1.18)	0.0002	1.10 (1.03–1.16)	0.002
Tobacco use	1.20 (1.03–1.40)	0.02	1.16 (1.00–1.34)	0.04
Heavy Alcohol Use	1.14 (1.05–1.24)	0.002	1.09 (1.00–1.18)	0.04

BCG, Bacillus Calmette–Guérin; RR, Risk Ratio.

^a^ Comorbid disease includes any of the following: heart disease, high blood pressure, asthma, kidney disease, use of steroids or chemotherapy or immunosuppressant, any other self-reported chronic illness.

**Table 3:** Univariate and multivariate analyses of baseline characteristics associated with incident tuberculosis (TB) infection.

	Person-years	Univariate HR (95% CI)	p value	Multivariate HR (95% CI)	p value
**Household Contacts Characteristics**					
Age Categories	3459				
< 5 years		0.25 (0.21–0.31)	<0.001	0.26 (0.20–0.33)	<0.001
5 to 11 years		0.31 (0.26–0.38)	<0.001	0.33 (0.26–0.42)	<0.001
12 to 19 years		0.37 (0.30–0.45)	<0.001	0.39 (0.30–0.49)	<0.001
20 to 49 years		0.89 (0.76–1.04)	0.13	0.86 (0.73–1.02)	0.09
≥50 years		Reference		Reference	
Male	3459	0.90 (0.82–1.00)	0.06	1.07 (0.96–1.21)	0.22
Nutritional Status	3459				
Underweight		0.60 (0.36–0.99)	0.05	0.56 (0.31–1.02)	0.06
Overweight		1.71 (1.54–1.90)	<0.001	1.09 (0.95–1.24)	0.21
Normal		Reference		Reference	
Comorbid Disease^a^	3459	1.34 (1.18–1.51)	<0.001	0.91 (0.79–1.06)	0.2
Number of BCG Scars	3459				
0		Reference		Reference	
1		1.51 (1.28–1.79)	<0.001	1.41 (1.17–1.69)	<0.001
2		3.00 (2.46–3.65)	<0.001	1.71 (1.37–2.14)	<0.001
> 2		3.11 (2.31–4.18)	<0.001	1.65 (1.19–2.29)	0.003
Isoniazid Preventive Therapy	3459	0.50 (0.44–0.57)	<0.001	NA^b^	
Heavy Alcohol Use	3408	1.59 (1.24–2.02)	<0.001	0.91 (0.70–1.19)	0.50
Tobacco use	3436	1.77 (1.41–2.21)	<0.001	1.15 (0.89–1.49)	0.28
HIV Positive	3422	0.67 (0.21–2.21)	0.52	0.49 (0.14–1.65)	0.25
Socioeconomic Status	3394				
Middle		Reference		Reference	
Low		1.00 (0.87–1.15)	0.98	1.01 (0.87–1.18)	0.89
High		1.30 (1.11–1.52)	0.001	1.12 (0.95–1.33)	0.18
Self-Reported Diabetes Mellitus	3441	1.74 (1.18–2.55)	0.005	0.83 (0.52–1.33)	0.44
**Index Case Characteristics**					
HIV Positive	3437	1.05 (0.73–1.50)	0.80	NA	
Smear Positive	3459	1.26 (1.08–1.46)	0.003	1.29 (1.09–1.52)	0.003
Cavitary Disease	3417	1.39 (1.22–1.58)	<0.001	1.30 (1.13–1.51)	<0.001
Tobacco use	3390	0.80 (0.51–1.25)	0.33	NA	
Heavy Alcohol Use	3334	1.15 (0.93–1.41)	0.19	1.22 (0.97–1.52)	0.09

BCG, Bacillus Calmette–Guérin; HR, Hazard Ratio.

^a^ Comorbid disease includes any of the following: heart disease, high blood pressure, asthma, kidney disease, use of steroids or chemotherapy or immunosuppressant, any other self-reported chronic illness.

^b^ Variable identified as a time-varying covariate.

**Table 4:** Body mass index (BMI) and risk of incident tuberculosis (TB) infection among household contacts over 19 years of age.

	Multivariate HR^a^ (95% CI) N = 2016	p value
BMI 18.5 to < 25 kg/m2	Reference	
BMI 25 to < 30 kg/m2	1.09 (0.93–1.28)	0.30
BMI 30 to < 35kg/m2	1.19 (0.96–1.48)	0.11
BMI ≥ 35 kg/m2	1.22 (0.89–1.67)	0.21
		p trend = 0.07

HR, Hazard Ratio.

^a^ Adjusted for age, sex, BMI categories, comorbid disease (heart disease, high blood pressure, asthma, kidney disease, use of steroids or chemotherapy or immunosuppressant, any other self-reported chronic illness), number of BCG scars, isoniazid preventive therapy, heavy alcohol consumption, tobacco use, HIV status, socioeconomic status, self-reported DM, index smear status, index cavitary disease and index heavy alcohol use.

### Risk of TB disease progression

**Fig 2:**
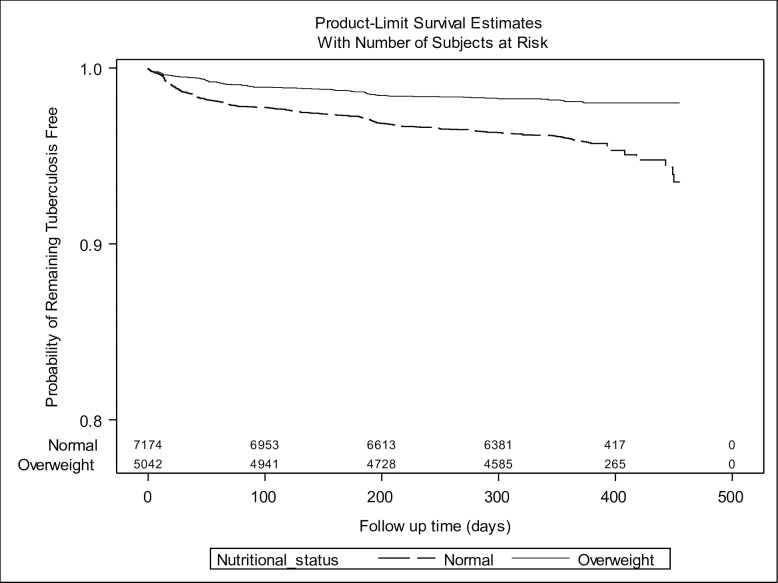
Kaplan-Meier survival plot of tuberculosis disease incidence stratified by nutritional status.

To assess the impact of being overweight on progression to TB disease, we excluded 218 contacts diagnosed with co-prevalent TB (Fig 1). Four hundred and six incident cases of secondary TB disease occurred during a total follow up time of 12,013 person-years, resulting in an incidence rate of 3380 per 100,000 person-years (95% CI 3051–3708). Among the secondary cases, 286 (70.4%) had PTB that was microbiologically confirmed, 72 (17.7%) were diagnosed with PTB by a physician based on the results of a chest x-ray or by clinical symptoms, and 42 (10.3%) had extra-pulmonary TB (EPTB). In the univariate analysis, overweight contacts were about half as likely to develop TB disease as those of normal weight (HR 0.47; 95% CI 0.37–0.59; p value <0.001) [Fig 2; Table 5], and this result remained almost unchanged when we adjusted for potential confounders (HR 0.48; 95% CI 0.37–0.64; p <0.001) [Table 5].

**Table 5:** Univariate and multivariate analyses of baseline characteristics associated with incident tuberculosis (TB) disease.

	Person-years	Univariate HR (95% CI)	p value	Multivariate HR (95% CI)	p value
**Household Contacts Characteristics**					
Age Categories	12,013				
< 5 years		1.25 (0.85–1.86)	0.26	1.55 (0.97–2.48)	0.06
5 to 11 years		1.00 (0.67–1.49)	0.99	1.24 (0.78–1.97)	0.37
12 to 19 years		1.72 (1.20–2.44)	0.003	1.69 (1.11–2.56)	0.01
20 to 49 years		1.15 (0.83–1.60)	0.39	1.15 (0.81–1.64)	0.44
≥50 years		Reference		Reference	
Male	12,013	1.18 (0.96–1.44)	0.11	1.16 (0.93–1.44)	0.19
Nutritional Status	12,013				
Underweight		1.18 (0.62–2.26)	0.61	1.42 (0.74–2.74)	0.29
Overweight		0.47 (0.37–0.59)	<0.001	0.48 (0.37–0.64)	<0.001
Normal		Reference		Reference	
Comorbid Disease^a^	12,011	1.03 (0.80–1.32)	0.81	1.18 (0.90–1.55)	0.22
BCG Scar	12,011	0.63 (0.49–0.81)	<0.001	0.67 (0.51–0.87)	0.003
Isoniazid Preventive Therapy	12,007	0.46 (0.34–0.62)	<0.001	NA^b^	
Heavy Alcohol Use	11,781	1.07 (0.70–1.65)	0.75	0.91 (0.55–1.49)	0.70
Tobacco Use	11,889	0.85 (0.54–1.32)	0.46	0.92 (0.56–1.50)	0.73
HIV Positive	11,868	2.23 (0.68–7.29)	0.18	2.15 (0.64–7.22)	0.22
Socioeconomic Status	11,701				
Middle		Reference		Reference	
Low		1.19 (0.92–1.54)	0.18	1.18 (0.91–1.53)	0.21
High		0.93 (0.68–1.26)	0.63	1.02 (0.74–1.39)	0.92
Self-Reported Diabetes Mellitus	11,927	1.79 (0.96–3.31)	0.07	2.30 (1.17–4.53)	0.02
History of TB	11,994	1.98 (1.48–2.66)	<0.001	1.81 (1.30–2.51)	<0.001
**Index Cases Characteristics**					
HIV Positive	11,891	0.71 (0.34–1.49)	0.37	NA	
Smear Positive	12,006	1.68 (1.24–2.27)	0.001	1.64 (1.19–2.25)	0.002
Cavitary Disease	11,843	1.38 (1.09–1.76)	0.009	1.32 (1.03–1.69)	0.03
Tobacco use	11,773	1.39 (0.74–2.63)	0.30	NA	
Heavy Alcohol Use	11,529	1.14 (0.80–1.63)	0.47	NA	

BCG, Bacillus Calmette–Guérin; HR, Hazard Ratio.

^a^ Comorbid disease includes any of the following: heart disease, high blood pressure, asthma, kidney disease, use of steroids or chemotherapy or immunosuppressant, any other self-reported chronic illness.

^b^ Variable identified as a time-varying covariate.

Given the altered clinical manifestations of TB in children, we considered the possibility that the impact of being overweight might differ between children and adults. When we stratified by age group, we found being overweight decreased the risk of incident TB disease among those aged 12 and older but not among children under 12 (*p*-value for interaction 0.05; Table 6). Among adults aged 20 and older, the risk of TB disease was dose dependent, with the hazard of TB disease declining with each BMI category compared to normal BMI: BMI 25 to < 30 kg/m^2^ (HR 0.45; 95% CI 0.32–0.63; p <0.001), BMI 30 to < 35kg/m^2^ (HR 0.37; 95% CI 0.22–0.63; p <0.001) and BMI ≥ 35 kg/m^2^ (HR 0.30; 95% CI 0.12–0.74; p 0.009) [Table 7; p trend <0.001]

**Table 6:** Nutritional status and risk of incident tuberculosis (TB) disease stratified by age.

	All secondary TB cases		Microbiologically confirmed secondary pulmonary TB cases	
Age Categories	Overweight vs. Normal Weight Multivariate HR^a^ (95% CI) N = 11,363	p value	Overweight vs. Normal Weight Multivariate HR^a^ (95% CI) N = 11,256	p value
<5 years	0.73 (0.32–1.63)	0.44	NA^b^	
5 to 11 years	1.16 (0.55–2.45)	0.70	0.97 (0.37–2.58)	0.96
12 to 19 years	0.23 (0.06–0.93)	0.04	0.14 (0.02–1.04)	0.05
20 to 49 years	0.47 (0.33–0.67)	<0.001	0.51 (0.35–0.75)	0.001
≥50 years	0.36 (0.20–0.67)	0.001	0.37 (0.19–0.70)	0.003

HR, Hazard Ratio.

^a^ Adjusted for age, sex, nutritional status, comorbid disease (heart disease, high blood pressure, asthma, kidney disease, use of steroids or chemotherapy or immunosuppressant, any other self-reported chronic illness), BCG scar, isoniazid preventive therapy, heavy alcohol consumption, tobacco use, socio-economic status, TB history, index smear status and index cavitary disease.

^b^ None of the overweight study participants in this age group developed microbiologically confirmed pulmonary TB disease.

**Table 7:** Body mass index (BMI) and risk of incident tuberculosis (TB) disease among household contacts over 19 years of age.

	All secondary TB cases		Microbiologically confirmed secondary pulmonary TB cases	
	Multivariate HR^a^ (95% CI) N = 6487	p value	Multivariate HR^a^ (95% CI) N = 6457	p value
BMI 18.5 to < 25 kg/m^2^	Reference		Reference	
BMI 25 to < 30 kg/m^2^	0.45 (0.32–0.63)	<0.001	0.46 (0.32–0.67)	<0.001
BMI 30 to < 35kg/m^2^	0.37 (0.22–0.63)	<0.001	0.40 (0.23–0.71)	0.002
BMI ≥ 35 kg/m^2^	0.30 (0.12–0.74)	0.009	0.37 (0.15–0.92)	0.03
		p trend <0.001		p trend <0.001

HR, Hazard Ratio.

^a^ Adjusted for age, sex, BMI categories, comorbid disease (heart disease, high blood pressure, asthma, kidney disease, use of steroids or chemotherapy or immunosuppressant, any other self-reported chronic illness), BCG scar, isoniazid preventive therapy, heavy alcohol consumption, tobacco use, HIV status, socioeconomic status, self-reported DM, TB history, index smear status, and index cavitary disease.

When we evaluated only household contacts diagnosed with incident TB disease at least 90 days after index case enrollment, we found even lower risk of TB disease among overweight contacts compared to normal weight (aHR 0.41; 95% CI 0.28–0.62; p < 0.001) [Table 8]. When we restricted our analysis to contacts with microbiologically confirmed PTB only, we found that the overall point estimate for the HR changed by less than 10% (aHR 0.46; 95% CI 0.34–0.63; p <0.001) and the differences between the results in adults and children persisted (*p*-value for interaction 0.06; Tables 6 and 7). Finally, when we considered the effect of BMI on the risk of EPTB for all contacts, we found that the point estimate for the effect of being overweight was still less than one but this difference did not reach statistical significance (HR 0.80; 95% CI 0.35–1.81).

**Table 8:** Sensitivity analysis of baseline characteristics associated with incident tuberculosis (TB) disease diagnosed at least 90 days after index case enrollment.

	Person-years	Univariate HR (95% CI)	p value	Multivariate HR (95% CI)	p value
**Household Contacts Characteristics**					
Age Categories	11,995				
< 5 years		0.78 (0.42–1.46)	0.44	0.86 (0.41–1.83)	0.70
5 to 11 years		0.75 (0.41–1.37)	0.34	0.90 (0.45–1.93)	0.78
12 to 19 years		2.21 (1.36–3.58)	0.001	2.14 (1.18–3.85)	0.01
20 to 49 years		1.07 (0.67–1.70)	0.77	1.29 (0.77–2.17)	0.33
≥50 years		Reference		Reference	
Male	11,995	0.95 (0.71–1.27)	0.71	0.95 (0.70–1.30)	0.75
Nutritional Status	11,995				
Underweight		1.05 (0.38–2.87)	0.93	1.31 (0.47–3.63)	0.60
Overweight		0.44 (0.31–0.62)	<0.001	0.41 (0.28–0.62)	<0.001
Normal		Reference		Reference	
Comorbid Disease^a^	11,993	1.28 (0.92–1.80)	0.15	1.41 (0.97–2.03)	0.07
BCG Scar	11,993	0.60 (0.42–0.85)	0.004	0.65 (0.44–0.95)	0.03
Isoniazid Preventive Therapy	11,989	0.77 (0.53–1.11)	0.16	NA^b^	
Heavy Alcohol Use	11,763	0.97 (0.51–1.87)	0.94	0.75 (0.36–1.59)	0.46
Tobacco Use	11,871	0.92 (0.49–1.71)	0.79	1.01 (0.51–2.00)	0.97
HIV Positive	11,851	3.15 (0.75–13.28)	0.12	2.51 (0.56–11.22)	0.23
Socioeconomic Status	11,684				
Middle		Reference		Reference	
Low		1.47 (1.04–2.07)	0.03	1.52 (1.07–2.17)	0.02
High		1.01 (0.65–1.56)	0.97	1.13 (0.72–1.75)	0.60
Self-Reported Diabetes Mellitus	11,910	2.06 (0.90–4.72)	0.09	2.92 (1.22–7.01)	0.02
History of TB	11,976	2.66 (1.80–3.92)	<0.001	2.71 (1.78–4.13)	<0.001
**Index Cases Characteristics**					
HIV Positive	11,873	0.61 (0.21–1.82)	0.38	NA	
Smear Positive	11,988	1.71 (1.10–2.66)	0.02	1.74 (1.10–2.76)	0.02
Cavitary Disease	11,826	1.26 (0.90–1.75)	0.18	1.18 (0.84–1.67)	0.34
Tobacco use	11,756	0.83 (0.29–2.38)	0.72	NA	
Heavy Alcohol Use	11,512	1.13 (0.69–1.88)	0.62	NA	

BCG, Bacillus Calmette–Guérin; HR, Hazard Ratio.^a^ Comorbid disease includes any of the following: heart disease, high blood pressure, asthma, kidney disease, use of steroids or chemotherapy or immunosuppressant, any other self-reported chronic illness.

^b^ Variable identified as a time-varying covariate.

## Discussion

In this study of the risk of TB infection and disease among household contacts of index TB patients, we confirmed the previously noted inverse association between BMI and risk of pulmonary tuberculosis and made three novel observations. First, we found that obesity continued to confer protection against TB at BMI levels associated with severe (or class II) obesity; adults with BMIs higher than 35 kg/m^2^ were even less likely to develop TB disease than overweight contacts. The protective effect of high BMI persisted after we adjusted for potentially confounding factors including socio-economic status, uptake of IPT and BCG vaccination and diabetes mellitus. Secondly, we found no significant difference in the incidence of TB infection in obese and normal household contacts thereby providing evidence that this protective effect is due to a reduction in disease progression rather than susceptibility to infection. Lastly, we addressed the question of whether high BMI protects against childhood TB and found no evidence for a protective effect of being overweight against either TB infection or disease in children under 12 years of age.

Our findings are quantitatively consistent with recent studies across various settings that have demonstrated an association between elevated BMI and decreased risk of TB disease. In a cohort of elderly persons in Hong Kong, Leung et al. found that obese individuals were about one third as likely to develop TB disease as were people of normal weight [11], and in the US NHANES study, Cegielski et al. found that obese adults were five-fold less likely to progress to TB disease than those of normal weight [13]. A similar decrease in risk of incident TB disease in obesity was also noted among HIV-infected adults in South Africa [12]. None of these studies, however, reported the risk of TB disease at very high BMIs, and it was thus unclear if obesity-associated immune modulation might have a detrimental effect at extreme BMI levels. We found no such decline in protection at BMIs over 35 kg/m^2^ although our population did not include enough individuals with morbid obesity (BMIs ≥ 40 kg/m^2^) to evaluate the trend at this level.

While previous studies have speculated that obesity reduced risk of TB disease progression rather than TB infection, only two published reports have examined infection by anthropometric status [8–9]. Palmer et al. followed over 60,000 US navy recruits aged 17–21 years from 1949 to 1955 measuring baseline TST and followed the cohort for TB disease. Although these investigators noted that lower height and increased weight were both associated with active TB disease, they found no difference in baseline TST reactivity by “body build” [8]. Edwards et al. reported similar results in a study of over 800,000 US navy recruits followed between 1958 and 1967 [9]. Because these studies measured body build and TST status only at baseline, it is not possible to determine the causal association between anthropometric measures and TST conversion. Nonetheless, these early observations are consistent with our finding that there was no association between baseline BMI and subsequent TB infection.

Previous studies on the association between BMI and TB disease have also focused on adult cohorts, and there is thus little evidence available on the association between BMI and TB risk in young children, despite the fact that obesity is increasingly common in this age group [27]. Our finding that the protective effect of BMI of TB disease observed in those aged 12 years of age or older was not present in younger children emphasizes the distinct clinical entity of childhood TB and raises the possibility that immunological correlates and mechanisms of protection in this group are markedly different from those in adults. Consistent with previous studies [10, 11, 28], we also found that the protective effect of high BMI appears to be specific to PTB. Given the immune profiles associated with EPTB differ from those associated with pulmonary disease [16, 17], this raises the question of whether those at risk for EPTB harbor a subtle immune defect that is not modified by diet induced adiposity.

How might high BMI protect against progression to TB disease, especially when it does not appear to confer this protection against other infectious diseases? After uptake of MTB bacilli in macrophages, MTB stimulates macrophage differentiation into lipid-laden foam cells through induction of intracellular lipid accumulation and reduced cholesterol efflux [29, 30]. This alteration in host macrophage metabolism allows MTB to use host lipids as a primary nutrient source and may facilitate a shift to a persistent “dormant” state that enables bacilli to survive within macrophages [31, 32]. Furthermore, human adipose tissue has been shown to provide a reservoir where MTB bacilli can survive in a non-replicating state [33, 34]. These data thus support the hypothesis that after TB infection, increased host adiposity could promote latency rather than progression to TB disease [7]. Other studies show that adipose tissue modulates adaptive immune responses through the production of adipocytokines such as leptin, which tends to increase with rising BMI [35]. Leptin not only regulates appetite through a direct impact on the hypothalamus but also stimulates the proliferation of naïve T cells and promotes T helper 1 (Th1) cytokine response [35, 36], both of which play critical roles in controlling MTB bacilli after host infection [37]. Leptin deficient mice have been shown to have increased susceptibility to PTB [38], and leptin levels have repeatedly been shown to be low in patients with TB compared to healthy controls [39, 40]. Nonetheless, few studies to-date have assessed the impact of high pre-existing leptin levels on TB risk or pathology. Notably, one recent study found that baseline elevation of circulating leptin contributes to the development of H1N1 influenza induced lung injury in mice, which improved upon administration of anti-leptin antibody [41]. While these findings may help explain why obesity increases the risk of influenza but not TB, studies of the impact of obesity or hyperleptinemia on TB infection in an animal model remain to be performed. Another mechanism by which obesity may protect against TB is suggested by the discovery that mycobacterial lipids function as antigens that form the specific targets of human αβ T cells [42]. CD1, a class of MHC I-like molecules expressed on the surface of human antigen-presenting cells activates mycobacterial lipid-specific T cells during human TB disease. Human serum lipids regulate CD1a, CD1b and CD1c expression on human dendritic cells through the PPAR-gamma signaling pathway [43], raising the possibility that nutritional status might control CD1-mediated T cell response to TB infection.

Our study has some notable limitations. First, we note that DM is a possible confounder of the relationship between high BMI and TB since DM risk increases with BMI and DM is a well-known risk factor for TB disease [44, 45]. Household contacts in our study were not routinely tested for hyperglycemia, and we therefore controlled for confounding by self-reported DM. A recent survey in Lima suggests that DM had been undetected in as many as 40% of those who met the diagnostic criteria for DM [22]. However, because we expect that more overweight and obese participants are likely to have undiagnosed DM than those of normal weight, this differential misclassification would have led us to underestimate the protective effect of increased BMI on TB disease risk in our study cohort. Future studies of risk factors for TB disease should ascertain presence of diabetes or chronic hyperglycemia with repeat plasma glucose levels or hemoglobin A1c. Second, given TB disease causes weight loss, contacts with subclinical TB that was undetected during baseline evaluation may have had lower BMI at enrollment; this may have led us to overestimate the observed effect of BMI on TB disease. To address this, we conducted a sensitivity analysis evaluating the impact of BMI on incident TB disease diagnosed at least 90 days after index case enrollment, and we found higher BMI was associated with even lower risk of TB disease diagnosed later during follow up. Third, some data suggest weight for height measures in Peruvian children may not be an accurate marker of fatty tissue content [46], and this may partially explain the lack of association between BMI and TB disease among children in our cohort. Furthermore, as noted above, our cohort included few people with BMIs over 40, and we were thus unable to determine the impact of further extreme levels of obesity.

## Conclusion

High BMI protects adults from TB disease progression even at BMIs over 35 kg/m^2^. This protection does not extend to TB infection and is not observed among children under 12 years of age.

## Supporting Information

S1 Dataset Data for prospective cohort study. (XLSX)
